# “West Foot Forward” – review of a high risk diabetes foot service

**DOI:** 10.1186/1757-1146-8-S2-O14

**Published:** 2015-09-22

**Authors:** Julia Firth, Michaela Barron

**Affiliations:** 1 Diabetes Foot Service, Western Health, Melbourne, Victoria, 3011, Australia

**Keywords:** Diabetes Foot Service outcomes

## Background

Diabetes in the Western Metropolitan Region has become a significant health issue. The most common complication of diabetes is serious foot disease leading to amputation. Literature supports the need for dedicated diabetes foot teams and that a multidisciplinary approach for patients with diabetic foot ulcers is effective in reducing the number of amputations. In 2010, senior medical and allied health clinicians proactively sought funding to address the gap in services available to meet the health care needs of patients with diabetes related foot complications.

## Process

Review of demographics, patient outcomes relating to service objectives were analysed from the period February 2011(inception of service) until May 2014.

## Findings

Objective 1: To provide timely, accessible and evidence based expert high risk foot care across the continuum of care

• Total of 497 admissions to the service with 72 current active patients

• 7246 episodes of care across the continuum of care

• The average length of stay in the service is 128 days with a range between 0-743 days

• average age of patients is 65yrs with 67% of patients male

• A low percentage of patients have been admitted multiple times to the program with the majority (83%) being single patient admissions

Pre-service implementation the average LOS was close to 16 days with a reduction to just under 10 days at the end of 2013.

Objective 2: To reduce the length of stay of inpatients with diabetes related foot complications

• Pre-service implementation the average LOS was close to 16 days with a reduction to just under 10 days at the end of 2013.

Objective 3: To improve clinical outcomes, such as amputation rates, for people with diabetes who have foot complications

• Below/above knee amputations have reduced by 44% and toe amputations have reduced by 56%.

Objective 4: To achieve successful treatment outcomes for patients with high risk foot complications

• 54% of patients completed treatment successfully

• 24% of patients completed treatment to a stage where they could be discharged to a community provider

• 7% of patients died during treatment due to extensive co-morbidities

• 6% of patients failed to attend appointments

## Conclusion and clinical relevance

The Diabetes Foot Service is providing a gold standard of care with an emphasis on excellence, patient focused outcomes, multidisciplinary service provision as well as contributing to the body of research in combating diabetes related foot disease.

